# Supplementary material for: A UMLS-based spell checker for natural language processing in vaccine safety
Source: BMC Med Inform Decis Mak. 2007 Feb 12;7:3. doi: 10.1186/1472-6947-7-3 (PMC1805499; doi:10.1186/1472-6947-7-3)
Supplement: Additional file 13 — RAP application directory. Sets up directory for RAP (RDF application for PHP) source code [file 1472-6947-7-3-S13.gz › rap/doc/tutorial/usingtheModelAPI.htm]

Using RAP's Statement-Centric MemModel API


### RDF API for PHP V0.9.1

# Using RAP's Statement-Centric MemModel API

This turorial is part of the RAP - Rdf API for PHP documentation.

  

Radoslaw Oldakowski, Daniel Westphal   
October 2004

  

This tutorial demonstrates how RAP's statement-centric MemModel API is used
for creating and manipulating RDF models.   
More information about the MemModel API is found in the MemModel PHPdocs.
An overview about RAP's other APIs is given in Introduction
to RAP.

### Contents:

|  |  |
| --- | --- |
| 1. | Installation |
| 2. | Creating Statements |
| 3. | Generating RDF Models |
| 4. | Printing Out a Model |
| 5. | Saving a Model Serialization to File |
| 6. | Blank Nodes |
| 7. | Typed Literals |
| 8. | Traversing a Model |
| 9. | Comparing and Combining Models |
| 10. | Querying a Model |
| 11. | Reification |
| 12. | Terminating a Model |
| 13. | Implementation Notes |

  
  

### 1. Installation

To get started we need to download the most recent version of RDF API for PHP
from http://sourceforge.net/projects/rdfapi-php/
and unzip the package. Note that RAP requires PHP 4.2 or later.

Next we can begin with an example script. The first step is to include all
RAP classes. We do it by defining the directory in which the API has been
unzipped and then include the file `RdfAPI.php` as shown below.

`define("RDFAPI_INCLUDE_DIR", "C:/Apache/htdocs/rdf_api/api/");
  
include(RDFAPI_INCLUDE_DIR . "RdfAPI.php");`

  
  

### 2. Creating Statements

Creating statements with RAP is extremely easy. Since each statement in RAP
is represented by an object (`Statement`), we have to create an
instance of this object with subject, property, and value passed as parameters
to the constructor function.

Consider the following statement written in N-Triple notation:
  
  
`<http://www.example.org/someDocument.html>
<http://www.purl.org/dc/elements/1.1/creator> "Radoslaw
Oldakowski".`  
  
In order to represent this statement in RAP we first
create its components (subject and predicate - both are resources indicated by
URIs), which we will also be using later on in this tutorial:

`$someDoc = new Resource
("http://www.example.org/someDocument.html");   
$creator = new Resource
("http://www.purl.org/dc/elements/1.1/creator");`

and then pass them together with the third component (object
`Literal`) to the constructor function:

`$statement1 = new Statement ($someDoc, $creator, new Literal
("Radoslaw Oldakowski"));`

RDF API for PHP also provides support for common vocabularies like Dublin Core used in this
statement. So instead of creating a new Resource (`$creator`)
representing the creator property we can also use the pre-defined variable
`$DC_creator` (see script DC.php).

In this straightforward way we have created our first object
`Statement`. Having this object, we could now compare it with other
statements (method `compare()`), serialize to string
(`toString()`), reify (`reify()` - see Section
11), or what we probably would do in most cases add it to a model.

  

### 3. Generating RDF Models

An overview of the different kinds of RAP models is found in Introduction
to RAP**.** RDF models can be stored either in main memory
(MemModel) or in a relational database (DbModel). Except for some indexing mechanisms
and Statement Iterator feature in the case of MemModel, all the other methods
are equal for both classes. So we can manipulate RDF models using the same function
names and expect the same output, regardless of the storage mechanism chosen.
In this tutorial I will focus on MemModels, whereas working with DbModels is
described here.  
The different kinds of models are created using a model factory.

In order to generate a new MemModel we simply write:

`$model1 = ModelFactory::getDefaultModel();`

To specify a *base URI* of a MemModel, we can either pass the URI string
as parameter to the constructor function or set it for an existing model using
the method `setBaseURI()`. In our example we just leave the default
setting and proceed with adding our first statement
(`$statement1`):

`$model1->add($statement1);`

In this simple way we have created a model containing one statement. However,
the method `add()` does not check if a MemModel already contains the
statement to be added. So if you want to avoid duplicates, use the method
`addWithoutDuplicates()` instead.

While generating a model we can add not only single statements but moreover
also entire models. To demonstrate this, we create a new MemModel containing two
statements:

`$model2 = ModelFactory::getDefaultModel()  
$model2->add(new
Statement($someDoc, new
Resource("http://www.example.org/myVocabulary/title"), new Literal("RAP
tutorial")));   
$model2->add(new Statement($someDoc, new
Resource("http://www.example.org/myVocabulary/language"), new
Literal("English")));`

and then add the second MemModel (`$model2`) just created to the
first one (`$model1`):

`$model1->addModel($model2);`

The parameter passed to the method `addModel()` can be an instance
of both classes MemModel and DbModel. If we now check the size of
`$model1` using the method `size()`:

`echo "\$model1 contains " .$model1->size() ." statements";`

we will see that `$model1` contains now three
statements:

**Output:**

$model1 contains 3 statements

  
  

### 4. Printing Out a Model

RDF API for PHP provides some useful methods for displaying the contents of a
MemModel. Using the method `writeAsHtmlTable()` we can print out the
MemModel as a table containing a set of triples. Methods `toString()`
and `toStringIncludingTriples()` create a plain text serialization of
the MemModel, whereas `writeRDFtoString()` and
`writeAsHtml()` serialize the model to RDF/XML. The latter converts
special chars to HTML entities, so that the serialization can properly be
displayed in a browser window.

Accordingly, the following piece of code:

`// Output $model1 as HTML table   
echo "<b>Output the
MemModel as HTML table: </b><p>";
  
$model1->writeAsHtmlTable();   
  
// Output the string
serialization of $model1   
echo "<b>Output the plain text
serialization of the MemModel: </b><p>";   
echo
$model1->toStringIncludingTriples();   
  
// Output the RDF/XML
serialization of $model1   
echo "<b>Output the RDF/XML
serialization of the MemModel: </b><p>";   
echo
$model1->writeAsHtml();`

would render this output in a browser window:

**Output:**

**Output the MemModel as HTML table:** 

|  |  |  |  |
| --- | --- | --- | --- |
| **Base URI:** | | | **Size:** 3 |
| **No.** | **Subject** | **Predicate** | **Object** |
| 1. | Resource: http://www.example.org/someDocument.html | Resource: http://www.purl.org/dc/elements/1.1/creator | Literal: Radoslaw Oldakowski |
| 2. | Resource: http://www.example.org/someDocument.html | Resource: http://www.example.org/myVocabulary/title | Literal: RAP tutorial |
| 3. | Resource: http://www.example.org/someDocument.html | Resource: http://www.example.org/myVocabulary/language | Literal: English |

  
  
**Output the
plain text serialization of the MemModel:**

MemModel[baseURI=; size=3]
Triple(Resource("http://www.example.org/someDocument.html"),
Resource("http://www.purl.org/dc/elements/1.1/creator"), Literal("Radoslaw
Oldakowski")) Triple(Resource("http://www.example.org/someDocument.html"),
Resource("http://www.example.org/myVocabulary/title"), Literal("RAP
tutorial")) Triple(Resource("http://www.example.org/someDocument.html"),
Resource("http://www.example.org/myVocabulary/language"),
Literal("English"))   
  
  
**Output the RDF/XML serialization of the
MemModel:**

<?xml version='1.0' encoding='UTF-8'?>  
<rdf:RDF  
   xmlns:rdf="http://www.w3.org/1999/02/22-rdf-syntax-ns#"  
   xmlns:rdfs="http://www.w3.org/2000/01/rdf-schema#"  
   xmlns:ns1="http://www.purl.org/dc/elements/1.1/"  
   xmlns:ns2="http://www.example.org/myVocabulary/">  
  
<rdf:Description rdf:about="http://www.example.org/someDocument.html">  
   <ns2:language>English</ns2:language>  
   <ns2:title>RAP tutorial</ns2:title>  
   <ns1:creator>Radoslaw Oldakowski</ns1:creator>  
</rdf:Description>  
  
</rdf:RDF>

  
  

### 5. Saving a Model Serialization to File

In addition to displaying a MemModel on the screen, we can save its
serialization to a file. RAP V0.8 supports the RDF/XML, N-Triple, and N3 syntax.
Thus, when we call the method `saveAs()` and pass the filename as the
first parameter, RAP will use the appropriate serializer according to the flag
(`'rdf'` ,`'n3' , or 'nt'`) provided as the second
parameter:

`$model1->saveAs("model1.rdf", "rdf");
  
$model1->saveAs("model1.n3", "n3");`

As output of the above code two files with RDF data have been created (model1.rdf
and model1.n3).
In the case that we leave out the second parameter, RAP will use the default
setting and will serialize the MemModel to RDF/XML.

To load an RDF model from a file we call the method `load()`
passing the name or a URL of the document containing RDF data and its
serialization syntax as parameters. If the second parameter is not provided, RAP
will decide upon the suffix which parser to use.

  

### 6. Blank Nodes

*Blank Nodes* in RDF API for PHP are represented by instances of object
`BlankNode`. Every B-Node is assigned a unique identifier which can
either be explicitly specified by the user or automatically generated for the
model passed to the constructor method. However, these identifiers have only a
local scope within a particular model. Thus, if two different RDF models use the
same *blankNodeID*, it does not necessarily mean that the Blank Nodes
identified by this ID must also be the same.

Consider the following modification of `$model1`: we replace the
literal `"Radoslaw Oldakowski"`, being an object in the first
statement, with a Blank Node. After that we make additional statements
describing this B-Node. To do this, we first create a new BlankNode allowing the
identifier to be automatically generated for our MemModel
`$model1`:

`$bNode = new BlankNode($model1);`

Next, using the method `replace()`, we exchange the objects:

`$model1->replace(NULL, NULL, new Literal("Radoslaw
Oldakowski"), $bNode);`

This will search for all statements having an object equal to the passed
literal and replace this literal with the value of the fourth parameter
(`$bNode`). Subsequently we add new statements describing the Blank
Node using RAP's pre-defined vCard vocabulary (`$VCARD_FN`,
`$VCARD_EMAIL`):

`$model1->add(new Statement($bNode, $VCARD_FN, new
Literal("Radoslaw Oldakowski")));   
$model1->add(new
Statement($bNode, $VCARD_EMAIL, new Literal("radol@gmx.de")));`

  
  

### 7. Typed Literals

The next statement we are about to add to `$model1` describes the
age of the person represented by the Blank Node `$bNode`. After
having created the literal object:

`$age = new Literal("26");`

we can explicitly indicate the datatype of the literal using the method
`setDatatype():`

`$age->setDatatype("http://www.w3.org/TR/xmlschema-2/integer");`

and then add the entire statement to the MemModel as usual:

`$model1->add(new Statement ($bNode, new
Resource("http://www.example.org/myVocabulary/age"), $age))`

Similarly, we can specify the language of a literal by calling the method
`setLanguage()` or by passing the language string as second parameter
to the constructor method on an object `Literal`.

  

### 8. Traversing a Model

If there is a need to traverse statements of a MemModel one by one, we can
use a Statement Iterator. The Statement Iterator is an object created by calling
the method `getStatementIterator()` on a MemModel, for instance:

`$it = $model2->getStatementIterator();`

Once instanced, the Statement Iterator can be ordered to return the current
(`current()`), next (`next()`), or previous
(`previous()`) statement. It can also move to a desired position
(`moveFirst()`, `moveLast()`, `moveTo()`). In
the following example we will use the iterator to output the label (URI or
literal string) of the subject, predicate and object of each statement in
`$model2`:

`while ($it->hasNext()) {   
     
$statement = $it->next();   
      echo
"Statement number: " . $it->getCurrentPosition() . "<BR>";
  
      echo "Subject: " .
$statement->getLabelSubject() . "<BR>";
  
      echo "Predicate: " .
$statement->getLabelPredicate() . "<BR>";
  
      echo "Object: " .
$statement->getLabelObject() . "<P>";   
}`

This will generate the following output on the browser
screen:

**Output:**

Statement number: 0   
Subject:
http://www.example.org/someDocument.html   
Predicate:
http://www.example.org/myVocabulary/title   
Object: RAP tutorial
  
  
Statement number: 1   
Subject:
http://www.example.org/someDocument.html   
Predicate:
http://www.example.org/myVocabulary/language   
Object: English

Since the Statement Iterator does not use the PHP's built-in array iterator,
we can independently use several instances of object StatementIterator on the
same model. This feature, however, is only accessible for instances of class
`MemModel`.

  

### 9. Comparing and Combining Models

RDF API for PHP provides three methods for comparing models. Using the
function `equals()` we can find out if two models are the same.
Methods `containsAny()` and `containsAll()` return a
corresponding Boolean value about statements shared by two models being
compared.

Furthermore, we can combine two different models by using either of the
methods `unite()`, `subtract()` or
`intersect()`, which will return a new MemModel respectively. More
details about these functions can be found in RAP
documentation.

As far as the methods described in this section are concerned, RAP offers a
high degree of flexibility by allowing the passed parameter to be an instance of
either a `MemModel` or `DbModel`.

  

### 10. Querying a Model

One of the probably most frequent activities performed on a model is querying
it. RDF API for PHP V0.9.1 offers various methods helping to find the desired
information. We can, for instance, take our `$model1` and search for
statements having subject `http://www.example.org/someDocument.html`.
For this purpose we use the method `find()` and pass the object
Resource `$someDoc` representing this particular subject as
parameter:

`$result = $model1->find($someDoc, NULL, NULL);
  
$result->writeAsHtmlTable();`

`NULL` will match anything. The value returned by this method is
another MemModel containing triples matching the passed statement pattern. If we
print out the result model as HTML table the following output will
occur:

**Output:**

|  |  |  |  |
| --- | --- | --- | --- |
| **Base URI:** | | | **Size:** 3 |
| **No.** | **Subject** | **Predicate** | **Object** |
| 1. | Resource: http://www.example.org/someDocument.html | Resource: http://www.purl.org/dc/elements/1.1/creator | Blank Node: bNode1 |
| 2. | Resource: http://www.example.org/someDocument.html | Resource: http://www.example.org/myVocabulary/title | Literal: RAP tutorial |
| 3. | Resource: http://www.example.org/someDocument.html | Resource: http://www.example.org/myVocabulary/language | Literal: English |

Besides the method find() which delivers a new MemModel as query result, RAP
also provides a Find Iterator for Traversing statements matching the specified
pattern. The Find Iterator is an object created
by calling the method `findAsIterator()` on
a `MemModel` and passing the statement pattern as parameter (similarly
like in the case of `find())`. Once instanced, the Find
Iterator can be ordered to return the current (`current()`)
or to find the next (`next()`) matching statement.

Methods `findFirstMatchingStatement()` and
`findCount()` are very similar to `find()`. The former returns
a model containing only one statement, whereas the latter merely returns the
number
of statements
matching the specified pattern.

If we want to be even more specific in our queries, we will be likely to use
the method `findRegex()`, which searches the model for triples
matching a Perl-style regular
expression. Furthermore, using the method `findVocabulary()` we
can filter all statements of a certain vocabulary.

RAP V0.9.1 also offers another method for querying RDF models using RDQL query
language. However, this method, as well as RDQL itself, is much more
sophisticated than the previously described functions and therefore is presented
separately in the RDQL
tutorial.

To increase query performance RDF API for PHP builds by default three search
indices over the subject, predicate, and object. Moreover, you can also
manually customize the indices used, according to the search pattern specified
in your query. This will result in much better performance (however, creating
an
index takes time). RAP allows users to build an index over the combined
labels of subject, predicate, and object or just subject and predicate, or
subject and object. To do this you simply have to call the method `index()`
and pass the appropriate parameter (`IND_SPO`, `IND_SP`, `IND_SO` respectively
or
`NO_INDEX` if you want to delete all indices).

`$model1 = ModelFactory::getDefaultModel();`  
`$model1->index(IND_SPO);`

### 11. Reification

To perform a reification in RAP, we simply call the method
`reify()` on an object `Statement`. The MemModel returned
will contain four statements according to the RDF
specification.

Moreover, we can use the method `reify()` on an instance of
`MemModel` or `DbModel` in order to reify all statements
of this particular model. To demonstrate this, we take our `$model2`:
and reify all its statements:

`$reified = $model2->reify();
  
$reified->writeAsHtmlTable();`

The corresponding result model `$reified` consists of 8 triples,
four for each reified statement as the browser output in form of an HTML table
shows:

**Output:**

|  |  |  |  |
| --- | --- | --- | --- |
| **Base URI:** | | | **Size:** 8 |
| **No.** | **Subject** | **Predicate** | **Object** |
| 1. | Blank Node: bNode1 | RDF Node: http://www.w3.org/1999/02/22-rdf-syntax-ns#type | RDF Node: http://www.w3.org/1999/02/22-rdf-syntax-ns#Statement |
| 2. | Blank Node: bNode1 | RDF Node: http://www.w3.org/1999/02/22-rdf-syntax-ns#subject | Resource: http://www.example.org/someDocument.html |
| 3. | Blank Node: bNode1 | RDF Node: http://www.w3.org/1999/02/22-rdf-syntax-ns#predicate | Resource: http://www.example.org/myVocabulary/title |
| 4. | Blank Node: bNode1 | RDF Node: http://www.w3.org/1999/02/22-rdf-syntax-ns#object | Literal: RAP tutorial |
| 5. | Blank Node: bNode2 | RDF Node: http://www.w3.org/1999/02/22-rdf-syntax-ns#type | RDF Node: http://www.w3.org/1999/02/22-rdf-syntax-ns#Statement |
| 6. | Blank Node: bNode2 | RDF Node: http://www.w3.org/1999/02/22-rdf-syntax-ns#subject | Resource: http://www.example.org/someDocument.html |
| 7. | Blank Node: bNode2 | RDF Node: http://www.w3.org/1999/02/22-rdf-syntax-ns#predicate | Resource: http://www.example.org/myVocabulary/language |
| 8. | Blank Node: bNode2 | RDF Node: http://www.w3.org/1999/02/22-rdf-syntax-ns#object | Literal: English |

  
  

### 12. Terminating a Model

If we know that we will no longer use an object `MemModel`, we
shall terminate it and free up resources held, especially while working with
MemModels containing a great amount of statements. We can do it by simply
calling the method `close()`. This way we will close all instances of
class `MemModel` used in this tutorial as follows:

`$model1->close();   
$model2->close();
  
$result->close();   
$reified->close();`

  

You can find the source code for this tutorial here.
A detailed description of all methods used can be found in the method
reference of class MemModel provided with RAP documentation.

### 13. Implementation Notes

The MemModel implementation stores statements in an array in the system memory.
If a new statement is added to the model, it's appended to the array and added
to a statement index in order to accelerate queries. MemModel is the fastest
of all Model API implementations.
